# The balance between Foxp3 and Ror-γt expression in peripheral blood is altered by tocilizumab and abatacept in patients with rheumatoid arthritis

**DOI:** 10.1186/s12891-016-1137-1

**Published:** 2016-07-16

**Authors:** Yoshifumi Tada, Nobuyuki Ono, Rie Suematsu, Satoko Tashiro, Yuri Sadanaga, Yukiko Tokuda, Yukihide Ono, Yoshinobu Nakao, Akihito Maruyama, Akihide Ohta, Syuichi Koarada

**Affiliations:** Department of Rheumatology, Saga University, 5-1-1 Nabeshima, Saga, 849-8501 Japan; Department of Nursing, Faculty of Medicine, Faculty of Medicine, Saga University, 5-1-1 Nabeshima, Saga, 849-8501 Japan

**Keywords:** Foxp3, Ror-γt, Abatacept, Tocilizumab

## Abstract

**Background:**

The balance between Th17 cells and regulatory T (Treg) cells has been shown to play an important role in the development of rheumatoid arthritis (RA). Recent studies have shown that treatment with abatacept (ABT) or tocilizumab (TCZ) affects Th17 and Treg cell populations. Although not unanimously accepted, several reports have shown that Treg cells are decreased by ABT and increased by TCZ, and that Th17 cells are decreased by TCZ. To further investigate the effects of ABT and TCZ on the skewing of T cell populations, we analyzed the expression of master regulators genes of helper T cell lineages following ABT/TCZ treatment of RA patients.

**Methods:**

Ten patients treated with ABT and 10 patients treated with TCZ were enrolled. Total RNA was extracted from peripheral blood cells at baseline, and after 12 and 24 weeks of therapy. The expression levels of T-bet, GATA3, Foxp3 and Ror-γt were semi-quantified using real-time PCR. The relative expression levels were expressed as the ratios of two genes (T-bet/GATA3, Foxp3/GATA3, Foxp3/T-bet, Foxp3/Ror-γt, Ror-γt/T-bet, Ror-γt/GATA3), and the changes in these ratios with treatment were determined.

**Results:**

The Foxp3/Ror-γt ratio was decreased after ABT therapy (0.67 ± 0.16 at 24 weeks, *P* = 0.0034) but was increased after TCZ therapy (2.00 ± 1.03 at 24 weeks, *P* = 0.0013). In addition, the Ror-γt/GATA3 ratio was decreased after TCZ therapy (0.78 ± 0.37 at 24 weeks, *P* = 0.0008). Except for these ratios, no significant skewing in the expression of these factors was detected. No significant relationship between clinical response to the treatment and change in the ratios of these factors was determined.

**Conclusion:**

Treatment with TCZ or ABT differently affected the balance between Foxp3 and Ror-γt expression in the peripheral blood of patients with RA.

**Electronic supplementary material:**

The online version of this article (doi:10.1186/s12891-016-1137-1) contains supplementary material, which is available to authorized users.

## Background

Rheumatoid arthritis (RA) is an autoimmune systemic inflammatory disease that affects joints and other tissues. The mechanisms of the pathology of RA are not well defined, although studies have shown that proinflammatory cytokines and various types of immune cells play significant roles in its development [1]. Recent therapies with biological agents have shown very good efficacy against RA. Tumor necrosis factor (TNF) α blockers inhibit the action of the major proinflammatory cytokine TNFα, resulting in the down-regulation of inflammatory cascades. On the other hand, the non-TNF blocking agents abatacept (ABT) and tocilizumab (TCZ) have different modes of action and might affect T cell activation and differentiation. ABT inhibits T cell activation by blocking the binding of the costimulatory molecule CD28 [2], whereas TCZ blocks interleukin (IL) -6 signaling that attenuates inflammatory cascades, and, in addition it might also affect T cell differentiation [3].

Various recent studies have shown that the balance between Th17 cells and regulatory T (Treg) cells plays a critical role in the development of RA [4]. Th17 cells secrete IL-17 that activates various cell types that are involved in the pathogenesis of RA and that are enriched in RA synovia [5, 6]. Treg cells interact with various cell types and regulate autoimmunity and inflammation; they are also an important player in the pathogenesis of RA [7]. Potential defects in Treg-mediated tolerance have been proposed in RA [8]. Based on these background findings, the changes in T cell subpopulations in patients with RA who were treated with ABT or TCZ have been investigated. Several reports showed that treatment with TCZ induced a decrease in Th17 cells [9, 10] and an increase in Treg cells [9, 11, 12], whereas a reduction in Treg cells has been reported after treatment with ABT [13, 14]. However these findings were not unanimous and conflicting results have been reported.

CD4+ helper T cell subsets express lineage-specific transcription factors, referred to as ‘master regulators’. Retinoic acid receptor-related orphan receptor-γt (Ror-γt) and forkhead box P3 (Foxp3) have been defined as the master regulators of Th17 cells and Treg cells, respectively [15, 16]. In this study, we investigated whether treatment with TCZ or ABT altered the relative expression levels of these master regulators in peripheral blood. We hypothesized that, if any change in helper T cell subpopulations is induced after biological therapy, the balance of the expression of master regulator genes may be altered. We focused in particular on the balance between Foxp3 and Ror-γt expression, corresponding to Treg cells and Th17 cells, respectively. Our results showed that the balance between Foxp3 and Ror-γt was differently regulated by TCZ and ABT in patients with RA.

## Methods

### Patients

Active RA patients who met the 1987 revised criteria of the American College of Rheumatology (ACR) for the classification of RA or the 2010 ACR/European league Against Rheumatism (EULAR) classification criteria [17, 18], and who were treated with TCZ or ABT were enrolled. This study was approved by the Saga University Hospital ethics committee (#2012-05-07), and written informed consent was obtained from all patients. Patients were intravenously treated with TCZ (8 mg/kg) every 4 weeks or with 500 mg of ABT on day 0, day 14 and day 28, and, thereafter, every 4 weeks.

### Clinical assessments

Clinical characteristics were obtained from medical records. Disease activity was assessed using the Clinical Disease Activity Index (CDAI) for patients treated with TCZ, and the Disease Activity Score for 28 joints (DAS28CRP) for patients treated with ABT.

### Real-time PCR

Blood samples were obtained at 0, 12 and 24 weeks after TCZ or ABT treatment. Peripheral blood was drawn into a PAXgene^TM^ RNA tube (PreAnalytiX GmbH, Switzerland) and was stored at -20 °C. Total RNA was extracted using the PAXgene Blood RNA kit (Qiagen) according to the manufacturer’s protocol, and was concentrated using the RNeasy MinElute Cleanup Kit (Qiagen). Total RNA (1 μg) was reverse transcribed using the Transcriptor First Strand cDNA Synthesis kit (Roche Diagnostics), and real-time PCR was performed using the LightCycler 480 with SYBR Green I master (Roche Diagnostics). The expression levels of the four master regulator genes, T-box transcription factor expressed in T cells (T-bet), GATA binding protein 3 (GATA3), Foxp3, and Ror-γt were determined. Primer sequences were as follows: for T-bet, 5′-CGGGAGAACTTTGAGTCC AT-3′ and 5′-CTGGGAACAGGATACTGGTTG-3′; for GATA3, 5′-GGCTCTACTACAAGCTTCACAATA-3′ and 5′-CGGGTTAAACGAGCTGTTCT-3′; for FoxP3, 5′-ATCCTGCCACCTGGAAGAAC-3′ and 5′-CCATCCTCCTTTCCTTGATCTTG-3′; for Ror-γt, 5′-CCAAGGCTCAGTCATGAGA-3′ and 5′-ACCCCTCACAGGTGATAAC-3′. GAPDH was used as a reference gene (Nihon gene research laboratories, Sendai, Japan). Normalized gene expression was derived from the ratio of the mRNA expression of the gene of interest to the GAPDH mRNA expression in each sample.

Because T cell numbers were different in each sample, the expression ratios of two genes (T-bet/GATA3, Foxp3/GATA3, Foxp3/T-bet, Foxp3/Ror-γt, Ror-γt/T-bet, Ror-γt/GATA3) were calculated for each point. The sequential changes in these ratios before and after treatment were determined (the ratios at baseline were defined as 1.00).

As controls, RNA samples from five healthy adults (four women and one man) were prepared, and real-time PCR was performed together with the samples from the RA patients.

### Statistical analysis

The Friedman test was used to compare the ratios of mRNA expression before and after therapy with the clinical indexes (CDAI and DAS28CRP). Spearman’s test was applied for correlations between clinical indexes and the changes in expression ratios. *P* < 0.05 was considered statistically significant. All statistical analyses were performed using Prism version 6 for Mac software (GraphPad, San Diego, USA).

## Results

### Patients and clinical response

The baseline characteristics of the patients are shown in Table 1. Ten patients were treated with ABT and an equal number of patients with TCZ. Parameters such as age, sex ratio, rheumatoid factor (RF), anti-cyclic citrullinated peptide (CCP) antibody positivity, concomitant methotrexate (MTX), and previous biologics usage, were not different between the two groups. Concomitant glucocorticoid was more frequent in the ABT group than in the TCZ group. In patients treated with ABT, the DAS28CRP score was decreased from 3.91 ± 1.00 before treatment to 2.74 ± 1.10 after 24 weeks of treatment (Table 1, *P* = 0.0179). According to the EULAR criteria, the number of patients with a good response, moderate response, and no response were three, four, and three, respectively. Three patients were in remission based on the DAS28CRP score. In the TCZ group, the CDAI was decreased from 16.5 ± 7.1 before treatment to 6.5 ± 5.3 after 24 weeks of treatment (Table 1, *P* = 0.0002). Eight patients showed a good response by EULAR criteria, and two patients were in remission based on the CDAI.

**Table 1:** Clinical characteristics at baseline and activity of rheumatoid arthritis before and after therapy

Patient characteristics	Abatacept group	Tocilizumab group
Number of patients	10	10
Age; yr, mean ± SD	59.8 ± 15.4	57.0 ± 14.0
Female, n (%)	8 (80)	7 (70)
RF positive, n (%)	9 (90)	9 (90)
Anti-CCP Ab positive, n (%)	9 (90)	9 (90)
CRP, mg/dl	2.7 ± 2.1	2.9 ± 3.0
MTX, n (%)	3 (30)	4 (40)
MTX dose, mg/week	10.7 ± 3.1	7.0 ± 2.6
Glucocorticoid, n (%)	9 (90)	3 (30)
Glucocorticoid dose, mg/day	6.3 ± 5.4	6.5 ± 4.1
Previous Biologics, n (%)	6 (60)	6 (60)
Infliximab, n (%)	1 (10)	
Etanercept, n (%)	4 (40)	3 (30)
Adalimumab, n (%)	2 (20)	1 (10)
Abatacept, n (%)		2 (20)
DAS28CRP, mean ± SD		
Before	3.91 ± 1.00	
After 24 weeks	2.74 ± 1.00	
CDAI, mean ± SD		
Before		16.5 ± 7.1
After 24 weeks		6.5 ± 5.3

*CCP* Cyclic citrullinated peptide, *CDAI* Clinical Disease Activity Index, *CRP* C-reactive protein, *DAS28* Disease Activity Score for 28 joints, *MTX* Methotrexate, *RF* Rheumatoid factor

### The expression ratios of master regulator genes

**Fig. 1:**
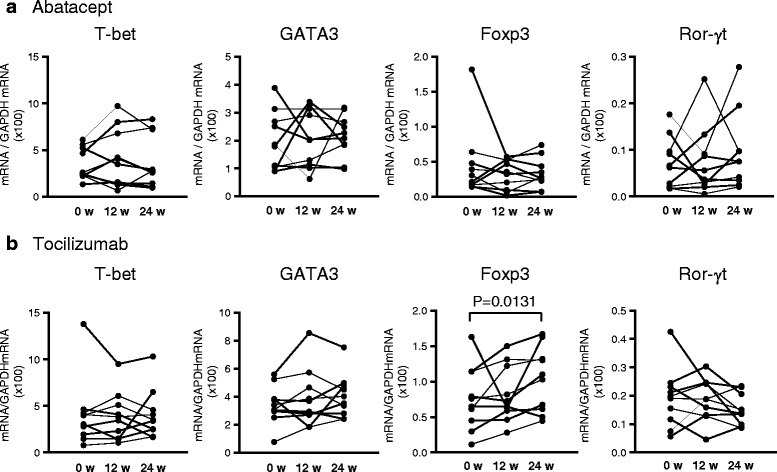
The expression levels of master regulator genes before and after biological therapy. **a**. Patients treated with abatacept. **b**. Patients treated with tocilizumab. In **a** and **b**, normalized gene expression was derived from the ratio of the mRNA expression of the gene of interest to the GAPDH mRNA expression

**Fig. 2:**
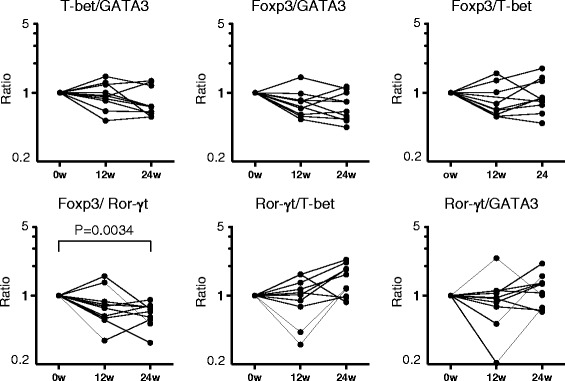
Expression ratios of master regulator genes in rheumatoid arthritis patients treated with abatacept. The ratios at baseline (0w) were defined as 1.00

**Fig. 3:**
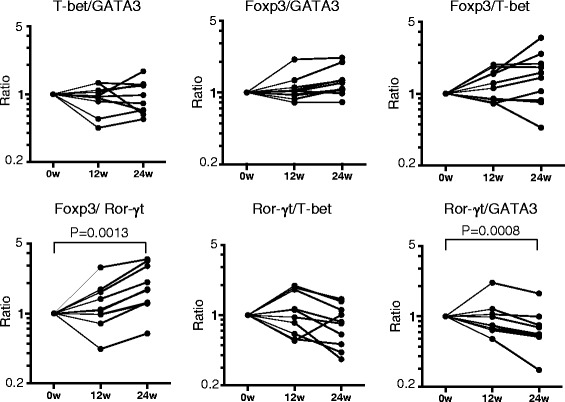
Expression ratios of master regulator genes in rheumatoid arthritis patients treated with tocilizumab. The ratios at baseline (0w) were defined as 1.00

We semi-quantified the expression of four master regulator genes: T-bet, GATA3, Foxp3, and Ror-γt, in peripheral blood using real-time PCR. Because the expression level of Ror-γt was very low and was undetectable when assaying a small amount of RNA, we prepared RNA from whole peripheral blood cells rather than purified T cells. The expression levels of master regulator genes before and after biological therapy were shown in Fig. 1. Foxp3 expression was increased after TCZ treatment (0.00757 ± 0.00459 before TCZ and 0.01028 ± 0.00454 at 24 weeks, *P* = 0.0131, Fig. 1b), whereas the expressions of Ror-γt by TCZ, and Foxp3 and Ror-γt by ABT were not significantly changed. The expression ratios of two genes were calculated, and the relative changes after ABT or TCZ treatment were determined. As shown in Fig. 2, the Foxp3/Ror-γt ratio was decreased after ABT therapy (0.83 ± 0.37 at 12 weeks and 0.67 ± 0.16 at 24 weeks, *P* = 0.0034). This ratio was decreased in eight of ten patients at 12 weeks, and in all patients at 24 weeks. Other ratios showed no significant changes. In patients treated with TCZ, in contrast to in patients treated with ABT, the Foxp3/Ror-γt ratio was increased after the therapy in all but one case at 24 weeks (Fig. 3, *P* = 0.0013). The relative ratio was 1.32 ± 0.67 at 12 weeks and 2.00 ± 0.97 at 24 weeks. In addition, the Ror-γt/GATA3 ratio was decreased after TCZ treatment (0.99 ± 0.45 at 12 weeks and 0.78 ± 0.37 at 24 weeks, *P* = 0.0008, Fig. 3). No significant skewing was detected in other ratios following treatment. The raw data are presented in Additional file 1.

The ratio of Foxp3/Ror-γt expression in peripheral blood cells from five age- and sex-matched healthy controls was compared to that of the RA patients before treatment, but no significant difference was observed (6.70 ± 2.23 vs, 6.59 ± 1.23). In addition, comparisons of other ratios of master regulator gene expression also showed no significant differences (Additional file 2: Figure S1).

The correlation between clinical response to ABT or TCZ and changes in the ratios of master regulators was analyzed. In the ABT group, the change in the Foxp3/Ror-γt ratio was not different between patients who achieved a good/moderate response and those who showed no response (0.63 ± 0.16 and 0.76 ± 0.15, respectively), and this ratio showed no correlation to the delta DAS28CRP score (0 and 24 weeks) (Additional file 2: Figure S2). Similarly, we found no relationship between the changes in the Foxp3/Ror-γt or the Ror-γt/GATA3 ratio and the delta CDAI (0 and 24 weeks) in patients treated with TCZ (Additional file 2: Figure S3). We further analyzed the correlation between these ratios and some clinical factors (i.e. sex, previous biologics, autoantibodies, and age). However no clear correlations were observed.

## Discussion

Several recent studies have shown that the frequency of Th17 cells and Treg cells in peripheral blood is altered by treatment with ABT or TCZ in patients with RA [9–14, 19–21]. Generally Th17 cells promote autoimmunity and inflammation, including RA [6, 22], whereas Treg cells play a role in maintaining immune responses and preventing autoimmunity [8, 23]. In addition these two cell types are closely related. Initial studies reported that these two cell subsets are reciprocally regulated and mutually exclusively differentiated [24]. However, recent studies reported the existence of Foxp3+ Treg cells that secrete IL-17 [25] and of Ror-γt + Th17 cells that differentiated from Foxp3+ Treg cells [26]. These data suggest that the development of these two subsets is less stringent than previously thought and that transition from Treg cells to Th17 cells may occur under some circumstances.

Unexpectedly we did not found any difference in the Foxp3/Ror-γt ratio between RA patients at baseline and healthy controls. Previous studies have shown that Th17 cells were increased and Treg cells were decreased in active RA patients (9, 10). However some studies showed no significant differences in the percentages of Th17 cells (11) or Treg cells (13). It may be helpful to directly compare the expression levels of master regulators and the percentages of the T cell subsets and examine whether both analyses correspond. This still remains to be addressed.

Regarding ABT treatment of RA patients, it has been shown that Treg cells are decreased after 3 or 6 months’ treatment [13, 14, 19]. On the other hand, Picchianti Diamanti A et al. reported that Treg frequency was not reduced but that function was recovered in patients with previous TNFα-blocking agent failure [20]. As for Th17 cells, a modest decrease was reported only in anti-CCP antibody-positive patients [14], or only after 12 months but not 6 months of treatment [19]. The combined data indicate that the main effect of ABT on the peripheral helper T cell population is to reduce Treg cells. Our result, that the Foxp3/Ror-γt ratio was decreased after ABT treatment is compatible with the results of those previous studies. The mechanism of the decrease in the Treg cell population is not clear; however, our results suggest that the down-regulation of Foxp3 expression by ABT leads to a reduction in Treg cells. It is of note that Foxp3/GATA3 and Foxp3/T-bet ratios showed no significant changes with treatment. These data suggested that ABT has different effects on the expression of Ror-γt, T-bet and GATA3, and that only Ror-γt expression is up-regulated. This effect may be due to reciprocal regulation of Ror-γt and Foxp3 and/or conversion of Foxp3^+^ Treg cells to Ror-γt^+^ Th17 cells [27]. ABT therapy induces a paradoxical phenomenon, i.e. Treg cells are decreased but inflammation is alleviated, which is in contrast to the effect of anti-TNFα and anti-IL-6 therapies [9, 12]. This phenomenon may be due to the critical role of the CD28-CD80/86 axis in the induction of Treg cells, and the functional substitution of Treg cells by ABT, a fusion protein of cytotoxic T lymphocyte-associated antigen 4 (CTLA4) and immunoglobulin G Fc chain, because the main machinery by which Treg cell exerts an inhibitory function is CTLA4 [28].

On the other hand, the effects of TCZ on T cell populations include an increase in Treg cells [9, 11, 12] and a decrease in Th17 cells [9, 10], although some investigators have reported that one or both of these populations were unchanged [11, 12, 21]. In our study, Foxp3 expression and the Foxp3/Ror-γt expression ratio was increased after TCZ therapy. This result is basically in line with previous cell population analyses. IL-6, together with TGF-β, induces the expression of Ror-γt and the generation of Th17 cells; at the same time, IL-6 inhibits TGF-β-induced Treg differentiation [29, 30]. It is therefore conceivable that a blockade of IL-6 signaling by TCZ attenuated the expression of Ror-γt and augmented the expression of Foxp3. Another interesting finding of our study is that there was a decrease in the Ror-γt/GATA3 ratio after TCZ therapy. Although a reciprocal relationship between Th2 cells and Th17 cells has not been established, TCZ therapy may up-regulate GATA3 expression and the Th2 population. Consistent with our result, Guggino et al showed an increase in Th2 (CD4^+^ IFN-γ^-^ IL-17^-^ IL-4^+^) cells after 3 months of therapy with TCZ [10].

In our study we did not find any relationship between clinical response to TCZ or ABT and change in the expression of master regulators. This finding may be due to the small sample size, and the small variance in clinical responses especially in patients treated with TCZ, who all showed a similar response. Alternatively, it is possible that the clinical response occurs at a time point after the alteration of master regulator gene expression. In other words, up-regulation or down-regulation of genes is induced by treatment with ABT or TCZ, but differences in subsequent events may modulate their effect. These subsequent events may include for example: modification of transcription factors, efficacy of induction of Th17 cells and Treg cells, or activation of these cells. Impaired function of Treg cells in RA, and its recovery after therapies with ABT or anti-TNF agents has been reported [13, 20, 30–32]. To address these issues, a further experiment using a larger population may be necessary.

Our study has some limitations. First, the expression of master regulator genes is not limited to CD4 + T cells. For example, it has been reported that Foxp3 is expressed in CD8+ T cells or B cells with regulatory properties and that these cells play a role in RA [33, 34]. Therefore the Foxp3 expression that we measured in our study may reflect not only Foxp3 expression in CD4+ T cells but also that in CD8+ T cells and B cells. Second, Th17 cells and Treg cells do not always strictly develop as discussed above, and the expression of the master regulator genes may not always correlate with the population and function of T cells. Treg cells that produce IL-17 have been identified in patients with RA [35]. Third, because of the limited numbers of patients, it was unable to compare the expression ratios in subpopulations. In addition to the response to the treatment as discussed, sexes, status of autoantibodies, and previous biologics may make differences.

## Conclusions

Our findings suggest that TCZ and ABT treatment modulates the expression of Foxp3 and Ror-γt in the peripheral blood. It is yet to be determined whether these changes directly lead to the induction of Treg cells and Th17 cells, but our data support the previous findings that the regulations of these cell populations may be one mode of action of TCZ and ABT.

## Abbreviations

ABT, Abatacept; ACR, American College of Rheumatology; CCP, Cyclic citrullinated peptide; CDAI, Clinical Disease Activity Index; CTLA4, Cytotoxic T lymphocyte-associated antigen 4; DAS28CRP, Disease Activity Score for 28 joints; EULAR, European league Against Rheumatism; Foxp3: Forkhead box P3; GATA3, GATA binding protein 3; IL, interleukin; MTX, Methotrexate; RA, Rheumatoid arthritis; RF, Rheumatoid factor; Ror-γt, Retinoic acid receptor-related orphan receptor-γt; T-bet, T-box transcription factor expressed in T cells; TCZ, Tocilizumab; TNF, Tumor necrosis factor; Treg, Regulatory T

## Additional files

Additional file 1: The expression levels of master regulator genes and clinical responses to ABT or TCZ. (XLSX 47 kb) Additional file 2:
**Figure S1**. Expression ratios of master regulator genes in rheumatoid arthritis patients before treatment and in control subjects. **Figure S2**. The correlation between Foxp3/Ror-γt ratio after 24 weeks of Abatacept treatment and delta DAS28CRP (0 and 24 weeks). **Figure S3**. The correlation between Foxp3/Ror-γt ratio or Ror-γt/GATA3 ratio after 24 weeks of Tocilizumab treatment and delta CDAI (0 and 24 weeks). (PDF 184 kb)
