# Supplementary material for: The balance between Foxp3 and Ror-γt expression in peripheral blood is altered by tocilizumab and abatacept in patients with rheumatoid arthritis
Source: BMC Musculoskelet Disord. 2016 Jul 16;17:290. doi: 10.1186/s12891-016-1137-1 (PMC4947268; doi:10.1186/s12891-016-1137-1)
Supplement: Additional file 2: — Figure S1. Expression ratios of master regulator genes in rheumatoid arthritis patients before treatment and in control subjects. Figure S2. The correlation between Foxp3/Ror-γt ratio after 24 weeks of Abatacept treatment and delta DAS28CRP (0 and 24 weeks). Figure S3. The correlation between Foxp3/Ror-γt ratio or Ror-γt/GATA3 ratio after 24 weeks of Tocilizumab treatment and delta CDAI (0 and 24 weeks). (PDF 184 kb) [file 12891_2016_1137_MOESM2_ESM.pdf]

# Figure S1

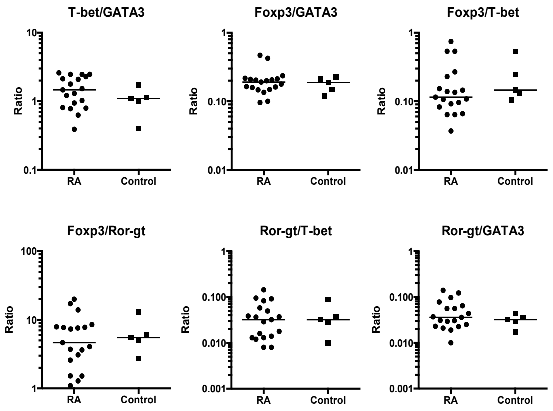


Additional file 2: Figure S1. Expression ratios of master regulator genes in rheumatoid arthritis patients before treatment and in control subjects.

Figure S2

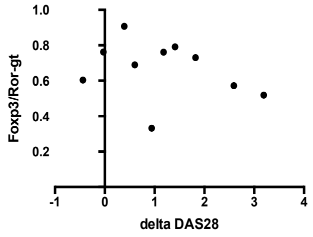


Additional file 2: Figure S2. The correlation between Foxp3/Ror-γt ratio after 24 weeks of Abatacept treatment and delta DAS28CRP (0 and 24 weeks).

Figure S3

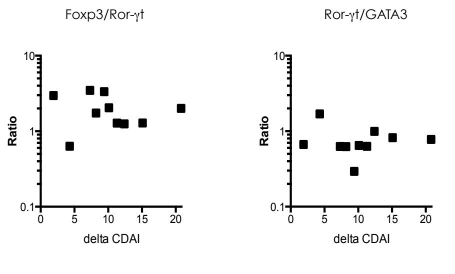


Additional file 2: Figure S3. The correlation between Foxp3/Ror-γt ratio or Ror-γt/GATA3 ratio after 24 weeks of Tocilizumab treatment and delta CDAI (0 and 24 weeks).
